# Fourier-based integration of quasi-periodic gait accelerations for drift-free displacement estimation using inertial sensors

**DOI:** 10.1186/s12938-015-0103-8

**Published:** 2015-11-23

**Authors:** Angelo Maria Sabatini, Gabriele Ligorio, Andrea Mannini

**Affiliations:** The BioRobotics Institute, Scuola Superiore Sant’Anna, Viale Rinaldo Piaggio, 34, 56025 Pontedera, Pisa Italy

**Keywords:** Gait analysis, Pose estimation, Fourier harmonic analysis, Inertial measurement unit

## Abstract

**Background:**

In biomechanical studies Optical Motion Capture Systems (OMCS) are considered the gold standard for determining the orientation and the position (pose) of an object in a global reference frame. However, the use of OMCS can be difficult, which has prompted research on alternative sensing technologies, such as body-worn inertial sensors.

**Methods:**

We developed a drift-free method to estimate the three-dimensional (3D) displacement of a body part during cyclical motions using body-worn inertial sensors. We performed the Fourier analysis of the stride-by-stride estimates of the linear acceleration, which were obtained by transposing the specific forces measured by the tri-axial accelerometer into the global frame using a quaternion-based orientation estimation algorithm and detecting when each stride began using a gait-segmentation algorithm. The time integration was performed analytically using the Fourier series coefficients; the inverse Fourier series was then taken for reconstructing the displacement over each single stride. The displacement traces were concatenated and spline-interpolated to obtain the entire trace.

**Results:**

The method was applied to estimate the motion of the lower trunk of healthy subjects that walked on a treadmill and it was validated using OMCS reference 3D displacement data; different approaches were tested for transposing the measured specific force into the global frame, segmenting the gait and performing time integration (numerically and analytically). The width of the limits of agreements were computed between each tested method and the OMCS reference method for each anatomical direction: Medio-Lateral (ML), VerTical (VT) and Antero-Posterior (AP); using the proposed method, it was observed that the vertical component of displacement (VT) was within ±4 mm (±1.96 standard deviation) of OMCS data and each component of horizontal displacement (ML and AP) was within ±9 mm of OMCS data.

**Conclusions:**

Fourier harmonic analysis was applied to model stride-by-stride linear accelerations during walking and to perform their analytical integration. Our results showed that analytical integration based on Fourier series coefficients was a useful approach to accurately estimate 3D displacement from noisy acceleration data.

## Background

In biomechanical studies Optical Motion Capture Systems (OMCS) are considered the gold standard for determining the orientation and the position (pose) of a human body part in a global reference frame [1]. However, the complexity of an OMCS, its cost and the limitations of using it outside the calibrated volume of the camera system has prompted research on the use of alternative sensing technologies; inertial sensors (a tri-axial accelerometer and a tri-axial gyroscope), integrated in an Inertial Measurement Unit (IMU) and attached to a human body part, are considered an appropriate choice in this regard [2].

The standard approach for estimating the pose of a body part from IMU signals involves three main steps [3]. First, the orientation is estimated by time-integrating the output of the tri-axial gyroscope from initial conditions that are computed using the specific force measured by the tri-axial accelerometer (roll and pitch angles, or attitude). For the computation of the initial conditions of attitude to be accurate, the body part of interest must be at rest, or be moving slowly; the initial condition of heading (yaw angle) cannot be determined unless, for instance, a tri-axial magnetic sensor is integrated in the IMU and the expression of the local magnetic field is known. The second step involves rotating the measured specific force based on the estimated orientation and compensating it for gravity, yielding the linear acceleration, also known as external acceleration, in the global reference frame. Finally, the linear acceleration is doubly time-integrated from the initial conditions of velocity and position which are assumed to be zero, yielding the three-dimensional (3D) displacement of the body part from the initial (generally unknown) absolute position. The whole process is commonly referred to as strap-down integration [4].

Strap-down integration is prone to several errors that tend to grow unbounded over time. These errors are due to the inertial sensors being affected by wideband measurement noise and bias that slowly evolves over time. Usually, sensor fusion methods for determining orientation are employed to mitigate the integration drift of gyroscopes. A wealth of literature is available to explain how to design sensor fusion methods, especially in difficult conditions when one or more of the following conditions recur: the body part is moving quickly, the magnetic environment is disturbed and/or the recording time is long [5]. There is comparatively less literature discussing the problem of doubly-time integrating the estimated linear acceleration to obtain accurate 3D displacement estimates [6].

In general, when no other aiding sensors are available to mitigate the integration drift of accelerometers, task-specific countermeasures should be considered. Task-specificity means that ad-hoc constraints of the motor task under investigation are exploited in the design of the pose estimation algorithm. For instance, in the development of pedestrian navigation systems, the dead-reckoning performance of on-foot IMUs can be greatly improved by so-called zero velocity updates [6, 7]. Another instance of task-specificity can be found in physiological tremor sensing or in studies of walking, where the patterns of motion are cyclical and the assumption of periodicity or quasi-periodicity is possible [8, 9]. In order to remove the integration drift from the estimated displacement, high-pass filtering can be applied by suitably choosing the cutoff frequency to separate cyclical components from non-cyclical components [10, 11]. Adaptive filtering algorithms based on truncated Fourier series have been proposed to detect periodic or quasi-periodic signals from a mixture of desired periodic signals and undesired signals. In particular, methods to obtain position information from periodic acceleration have been based on analytical integration so as to avoid drift caused by numerical integration [12, 13]. In a similar vein, orientation information was obtained through analytical integration of the measured angular velocity from a gyroscope during treadmill walking [14].

A stride-based approach to integrating periodic or quasi-periodic signals is to identify individual strides in preparation for stride-by-stride integration. The time functions of the linear acceleration over individual strides were mean subtracted in [9], where the canter locomotion of horses was studied. The time interval used for mean subtraction was chosen as a trade-off between minimizing the accumulation of numerical integration errors and capturing features that extend over a longer period of time (non-cyclical components)—a window of one stride to each side of the current stride was chosen for mean subtraction. Time integration was performed twice using the trapezoidal rule, with mean subtraction performed at each step of integration. A similar approach was pursued by [15] for the purpose of analyzing the vertical movement of the human body center of mass during walking; the main differences were the use of quaternions (rather than Euler angles) to describe the orientation and the method for dedrifting the estimated displacement, which was based on three-point windowing followed by third-order polynomial smoothing.

In this paper, we report and evaluate a novel stride-based approach that allows the 3D displacement of an IMU during cyclical patterns of motion to be determined. As our case study, we considered the lower trunk motion during walking. Our method is based on the analytical integration of linear acceleration over individual strides using Fourier harmonic analysis methods, which differs from the numerical integration and dedrifting approaches proposed in [9, 15]; in common with [9] the prerequisite for our method of work is that the individual strides can be identified.

Fourier harmonic analysis is the core of several approaches to gait assessment of elderly and pathologic subjects [16, 17, 18]. In our current research [19], the stride-by-stride Fourier harmonic analysis allows the harmonic ratio (HR) of lower trunk acceleration to be computed, e.g., [16, 20]. At the expense of a slightly increased computational workload, the proposed method may add new dimensions to the parameter space including HRs, stride time and variability by delivering estimates of linear velocity (not analyzed in this paper) and of 3D displacement; the proposed method offers thus promise, for instance, when applied to the study of the motion of the body center of mass [21, 22], or in quantifying external work [23], especially in those experimental settings where the use of an OMCS is difficult, e.g., studies of over-ground walking that require the acquisition of many strides.

Although this is not the envisaged application, treadmill walking of healthy subjects is the framework of validation in this paper. The lower trunk 3D displacement estimated using IMU signals was compared to the estimate obtained from an OMCS. Six different approaches to strap-down rotation, stride segmentation and time integration were considered for the purpose of displacement estimation. For each algorithmic variant the limits of agreement with the OMCS data were computed, based on procedures devised for method comparison studies [11, 24].

## Methods

### Wearable sensor system

The experiments described in this paper were performed using a wearable sensor system composed of two custom-made battery-powered wireless devices, named Wearable IMUs (WIMUs). In the current implementation each WIMU embeds a digital tri-axial gyro (InvenSense ITG-3200, with range ±2000°/s), a digital tri-axial accelerometer (Bosch BMA180, with range ±4 *g*, where *g* = 9.81 m/s^2^ is the gravity acceleration), a digital tri-axial magnetic sensor (Honeywell HMC5843), and a digital barometric altimeter (Bosch BMP085). Each WIMU is endowed with a 32-bit ARM Cortex processor (NXP Semiconductors LPC1768) and a Bluetooth (BT) transceiver for data communication with a host computer. A graphical user interface developed in Visual Basic 2008 makes it possible to configure the acquisition parameters, log WIMU sensor data and visualize their time plots on-line. WIMU sensor data were sampled at 100 Hz and digitally filtered on-board using a Butterworth second-order low-pass filter (cut-off frequency: 25 Hz). The WIMU sensor data were uploaded to the host computer via BT for further processing with customized functions using MATLAB software (The MathWorks Inc., Natick, MA, USA). Magnetic sensor and barometric altimeter data were acquired, however they were not used to run the computational procedures in this paper.

### Experimental protocol

Nine healthy subjects (5 males and 4 females) participated in the experiments after being informed about the nature and goals of the experimental procedures and after their consent had been provided. Age ranged from 24 to 54 years (29.8 ± 7.8 years), body mass from 52 to 87 kg (66.1 ± 10.8 kg), and height from 1.60 to 1.87 m (1.72 ± 0.08 m). Subjects were instructed to perform 2-min walking trials on a treadmill, after allowing them to familiarize with treadmill walking; five trials, each at different speeds, from 3 to 7 km/h at steps of 1 km/h, were performed by each subject. Care was taken that a rest period of 5 s with the subjects standing still in their upright posture preceded the start of each walking trial.

**Fig. 1:**
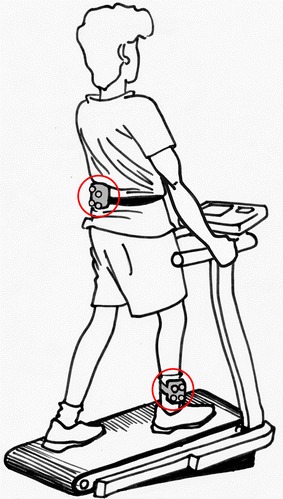
Experimental set-up showing a subject walking on the treadmill walking machine, instrumented with the WIMUs on the shank and the lower trunk

One WIMU was attached to the level of the fifth lumbar vertebra and secured with an elastic Velcro strap to keep it firmly in place (lower trunk WIMU); another WIMU was attached to the lateral aspect of the right shank (above the lateral malleolus) and secured with an elastic Velcro strap (shank WIMU), Fig. 1.

A five-camera OMCS (Bonita B10, Vicon Motion Systems Ltd., Oxford, UK) was used to acquire the reference kinematic data for the lower trunk WIMU. Four retro-reflective markers were mounted on a small plastic support rigidly attached to the WIMU case; marker trajectories were tracked by the OMCS at a rate of 100 samples/s and forward–backward filtered via a Butterworth second-order low-pass filter with a cut-off frequency of 12 Hz. Before performing integration, the acceleration signals were forward–backward filtered with the same filter used for the OMCS data.

### Inertial sensor calibration

The inertial sensors of the lower trunk WIMU and the tri-axial gyroscope of the shank WIMU were calibrated before each experimental session [5]. Before being worn, the lower trunk tri-axial accelerometer was calibrated using a standard least-squares method for estimating bias and scale factor along each axis [25]. The bias of the gyroscopes was captured with the WIMUs in place, during the rest period at the beginning of each walking trial, and then subtracted from the angular velocities measured during the walking trial. No specific calibration procedure was implemented to estimate the scale factor of the tri-axial gyroscopes.

### Reference frames

**Fig. 2:**
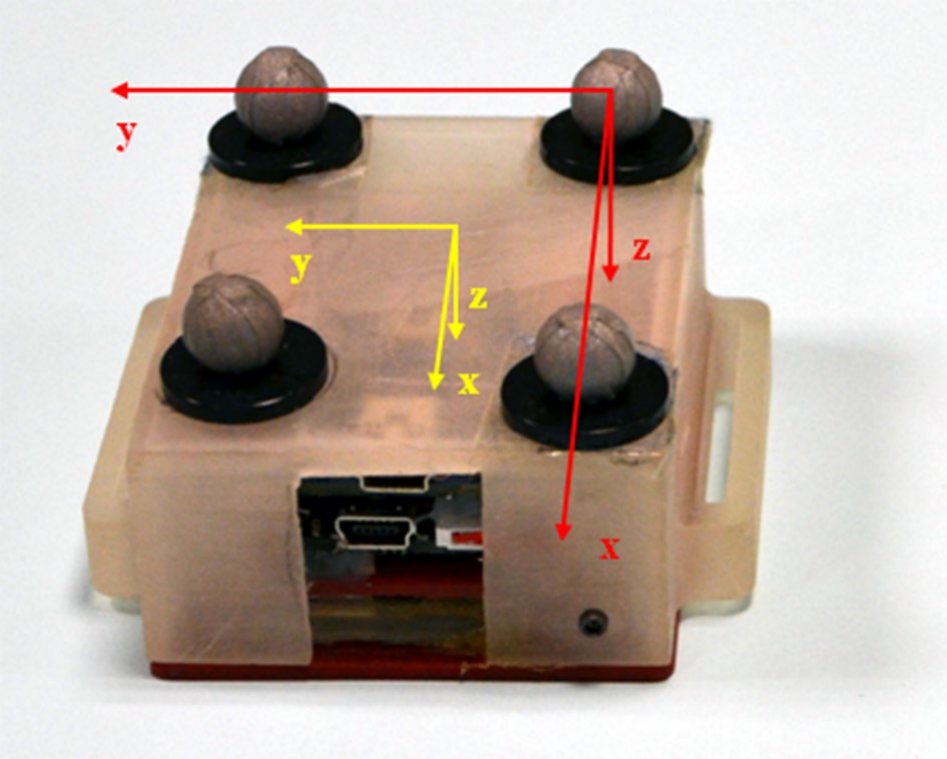
The plastic support to which the retro-reflective markers and the WIMU (underneath the cover) were attached. The frame in *red* was the MLF, the frame in *yellow* was the ULF

We denoted the Cartesian coordinate systems fixed with the WIMU and the OMCS, respectively, as the Unit Local Frame (ULF), and the Global Earth-fixed Frame (with one axis aligned with Gravity) (GGF). The X and Y-axes of the GGF were longitudinally and transversally oriented relative to the treadmill frame, respectively. A Marker-cluster Local Frame (MLF) was defined using the cluster of the four retro-reflective markers, so as to obtain its reference orientation with respect to the GGF, Fig. 2.

The time invariant orientation of ULF relative to MLF was estimated as described in [26]. The two local frames were computationally aligned and rotated so as to have one axis aligned with gravity during the rest period, at the beginning of each walking trial. Since the 3D orientation of ULF and MLF was expressed with respect to the same GGF, the orientation change of the lower trunk WIMU was assessed with respect to its own initial orientation. The orientation of the WIMU axes were X: Antero-Posterior (AP) and positive forward; Y: Medio-Lateral (ML) and positive to the right; Z: VerTical (VT) aligned with the direction of gravity and positive downwards. The reference quaternion from the MLF to the GGF was computed by applying standard methods to the marker trajectories [27].

For each WIMU, a virtual marker was created approximately in correspondence of the center of the tri-axial accelerometer chip, where the ULF origin was located, by using the positions of the four retro-reflective markers. The 3D displacement of the virtual marker at the lower trunk WIMU was the reference against which to compare the 3D displacement of the ULF origin estimated using the computational procedures explained below.

In order to synchronize OMCS and WIMU data streams, the angular velocity was estimated by differentiating the orientation computed from the OMCS marker positions. The cross-correlation between the estimated and the measured angular velocity was then computed and the lag existing between the two data streams was corrected.

### Computational procedures

The Extended Kalman Filter (EKF) developed in [5] was used to estimate the quaternion from the lower trunk ULF to the GGF. The main difference between the current implementation and the one in [5] was that, in the present application, the magnetic sensor measurements were not considered in the EKF measurement equations; the extra-state components of the state vector needed for self-compensation of magnetic disturbances and gyroscope bias were also dismissed. The estimated quaternion was used to perform the strap-down rotation of the measured specific force, so as to obtain the gravity-compensated expression of the lower trunk linear acceleration resolved in the GGF. Unless otherwise stated, the ML component of the angular velocity measured by the shank tri-axial gyroscope was used to determine the beginning of each stride [28, 29]. Alternatively, the beginning of each stride was determined using the lower trunk tri-axial accelerometer [10].

Stride-by-stride linear acceleration data were submitted to Fourier analysis. Specifically, each linear acceleration component (namely: ML, VT, AP) of the *i*-th stride was analyzed and the harmonic coefficients were computed up to a specified order *N* (*N* = 20):

1 $$\begin{array}{*{20}c} {acc_{\text{ML}}^{i} = a_{{{\text{ML}}i}} (0) + \mathop \sum \limits_{k = 1}^{N} \left( {a_{{{\text{ML}}i}} \left( k \right)\cos \frac{2\pi k}{{T_{i} }}t + b_{{{\text{ML}}i}} (k)\sin \frac{2\pi k}{{T_{i} }}t} \right)} \\ {acc_{\text{VT}}^{i} = a_{{{\text{VT}}i}} (0) + \mathop \sum \limits_{k = 1}^{N} \left( {a_{{{\text{VT}}i}} \left( k \right)\cos \frac{2\pi k}{{T_{i} }}t + b_{{{\text{VT}}i}} (k)\sin \frac{2\pi k}{{T_{i} }}t} \right)} \\ {acc_{\text{AP}}^{i} = a_{{{\text{AP}}i}} (0) + \mathop \sum \limits_{k = 1}^{N} \left( {a_{{{\text{AP}}i}} \left( k \right)\cos \frac{2\pi k}{{T_{i} }}t + b_{{{\text{AP}}i}} (k)\sin \frac{2\pi k}{{T_{i} }}t} \right)} \\ \end{array}$$

where $T_{i}$ is the duration of the *i*-th stride. The analytical integration of (1) where $a_{{{\text{ML}} i}} \left( 0 \right), a_{{{\text{VT}} i}} \left( 0 \right)$ and $a_{{{\text{AP}} i}} \left( 0 \right)$ were set to zero (equivalent to detrending a constant term from the original stride linear acceleration data) leading to the following expression of the mean-subtracted *i*-th stride velocity data:

2 $$\begin{array}{*{20}c} {vel_{\text{ML}}^{i} = \frac{{T_{i} }}{2\pi }\mathop \sum \limits_{k = 1}^{N} \frac{1}{k}\left( {a_{{{\text{ML}} i}} \left( k \right)\sin \frac{2\pi k}{{T_{i} }}t - b_{{{\text{ML}} i}} (k)\cos \frac{2\pi k}{{T_{i} }}t} \right)} \\ {vel_{\text{VT}}^{i} = \frac{{T_{i} }}{2\pi }\mathop \sum \limits_{k = 1}^{N} \frac{1}{k}\left( {a_{{{\text{VT}} i}} \left( k \right)\sin \frac{2\pi k}{{T_{i} }}t - b_{{{\text{VT}} i}} (k)\cos \frac{2\pi k}{{T_{i} }}t} \right)} \\ {vel_{AP}^{i} = \frac{{T_{i} }}{2\pi }\mathop \sum \limits_{k = 1}^{N} \frac{1}{k}\left( {a_{{{\text{AP}} i}} \left( k \right)\sin \frac{2\pi k}{{T_{i} }}t - b_{{{\text{AP}} i}} (k)\cos \frac{2\pi k}{{T_{i} }}t} \right)} \\ \end{array}$$

Finally, the expression of the mean-subtracted *i*-th stride displacement data follows:

3 $$\begin{array}{*{20}c} {displ_{\text{ML}}^{i} = - \left( {\frac{{T_{i} }}{2\pi }} \right)^{2} \mathop \sum \limits_{k = 1}^{N} \frac{1}{{k^{2} }}\left( {a_{{{\text{ML}}i}} \left( k \right)\cos \frac{2\pi k}{{T_{i} }}t + b_{{{\text{ML}}i}} (k)\sin \frac{2\pi k}{{T_{i} }}t} \right)} \\ {displ_{\text{VT}}^{i} = - \left( {\frac{{T_{i} }}{2\pi }} \right)^{2} \mathop \sum \limits_{k = 1}^{N} \frac{1}{{k^{2} }}\left( {a_{{{\text{VT}}i}} \left( k \right)\cos \frac{2\pi k}{{T_{i} }}t + b_{{{\text{VT}}i}} (k)\sin \frac{2\pi k}{{T_{i} }}t} \right)} \\ {displ_{\text{AP}}^{i} = - \left( {\frac{{T_{i} }}{2\pi }} \right)^{2} \mathop \sum \limits_{k = 1}^{N} \frac{1}{{k^{2} }}\left( {a_{{{\text{AP}}i}} \left( k \right)\cos \frac{2\pi k}{{T_{i} }}t + b_{{{\text{AP}}i}} (k)\sin \frac{2\pi k}{{T_{i} }}t} \right)} \\ \end{array}$$

The Fourier analysis was performed using the methods of functional data analysis and the MATLAB toolbox developed in [30].

A fundamental prerequisite for a correct harmonic analysis is that the samples, equally spaced in time, of the curve submitted to analysis exactly fits one walking cycle [31]. This circumstance did not necessarily occur in our data. Part of the problem was related to the accuracy in determining the beginning of each stride. Leaving the problem of inserted and missed gait cycles aside [28] (they never occurred during the experiments of this paper), the detection of heel strike (considered in our approach as the gait events signaling the beginning and end of each stride of the instrumented leg) was affected by some random error. Moreover, the linear acceleration estimate was affected by errors in determining the quaternion needed for the strap-down rotation. This means that each stride curve *f*(*t*) submitted to Fourier analysis generally does not comply with the requirement of being cyclical, namely it is not guaranteed that *f*(0) = *f*(*T*), where *T* is the stride duration.

**Fig. 3:**
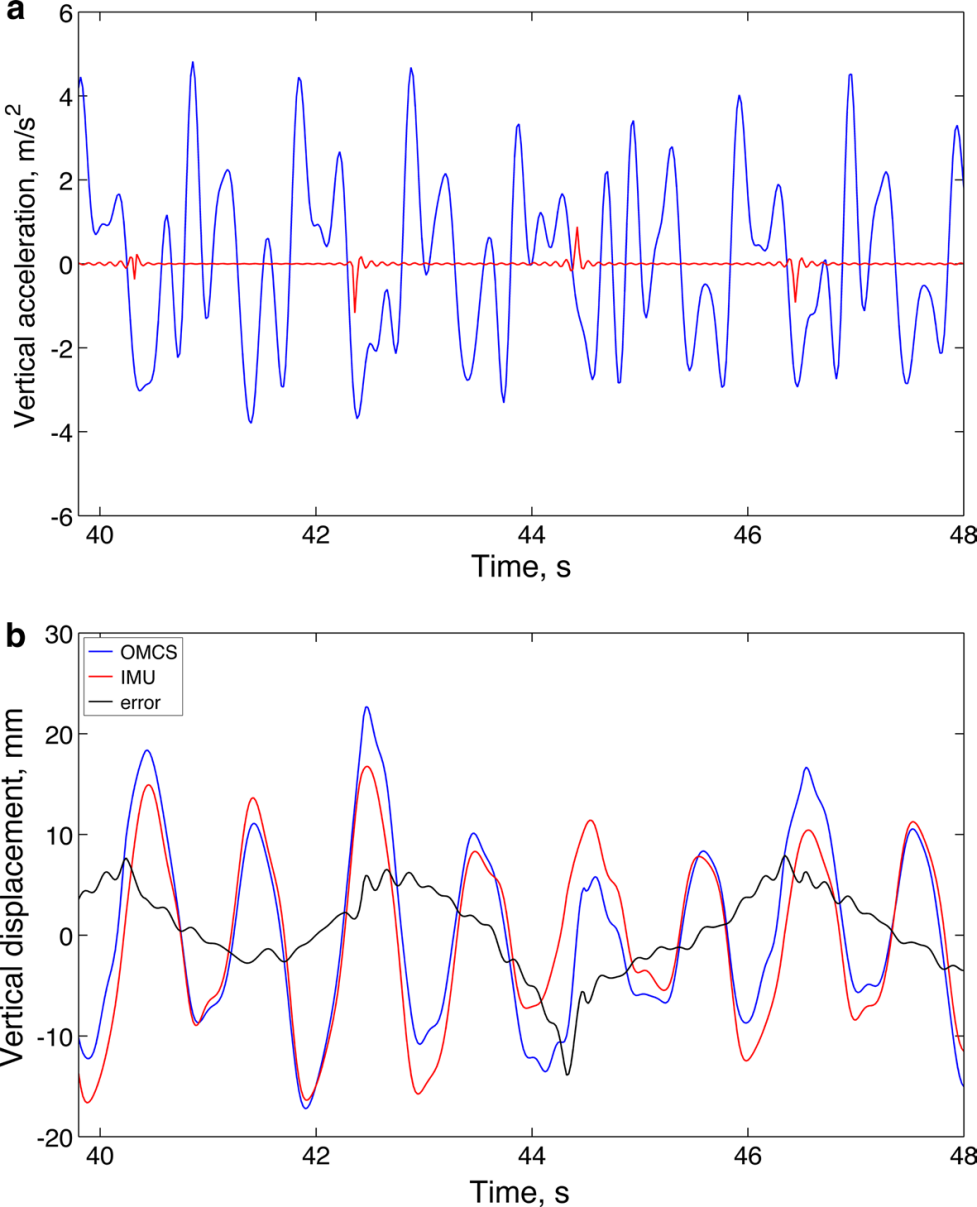
**a** Time functions of the vertical acceleration (*blue*) and the reconstruction error (*red*) for a Fourier partial series of 20 terms are shown superimposed (subject #1, walking speed: 5 km/h); **b** Time functions of the vertical displacement estimated by OMCS and by the proposed Fourier-based method, together with the time function of the difference. **a** shows the jumps occurring at the detected stride times and the oscillations due to Gibbs phenomenon on either side of the boundary between successive gait strides. **b** shows the mild effects of discontinuity jumps due to spline interpolation across boundaries and the presence of small-amplitude Gibbs oscillations on the estimated VT displacement

An interesting property of Fourier series near discontinuities arising from the condition *f*(0) = *f*(T) not being true is the Gibbs phenomenon. This phenomenon refers to the manner in which the Fourier series for a periodic function overshoots the values of the function on either side of a discontinuity [32]. The overshoots do not die out when the number of terms *N* retained in the Fourier expansion is increased. It turns out that as long as *N* is large, the height of the overshoot is about 9 % of the jump in the function, independent of the large number *N*. Figure 3 shows a representative example from our dataset. Superimposed on the plot of the vertical acceleration, we report the reconstruction error arising from a stride-by-stride Fourier analysis based on *N* = 20 terms in the partial series. It is noted that the frequency of the Gibbs oscillations depends on both *T* and *N*. In our dataset, frequency values are in the interval 10–25 Hz; hence, even for largest discontinuity jumps, the Gibbs oscillations of acceleration give rise to maximum displacement errors of some tenths of a millimeter, which in the present context are negligible compared to other error sources.

Since for cyclical movements the linear acceleration components would have zero-mean value [9], we dismissed the constant terms when performing the analytical integration. A check was also carried out to assess the accuracy of the reconstruction process in terms of the Root Mean Square Difference (RMSD) between $g\left( t \right)$ and the function $\hat{g}\left( t \right)$ reconstructed via the inverse Fourier series, normalized to the root mean square value of $g\left( t \right).$ Usually, normalized RMSD values lower than 5 % were achieved, which was deemed adequate for our purposes. The reference OMCS stride displacement data were also processed using stride-based piecewise-constant trend removal.

Since the reconstructed strides do not generally satisfy the continuity constraint at the boundary between consecutive strides, spline interpolation was applied to the concatenated stride traces for its ability to smooth discontinuities, yielding the entire trace of IMU and OMCS displacement data. The differences between peak and trough of the OMCS displacements, namely their peak-to-trough values, were also computed.

**Fig. 4:**
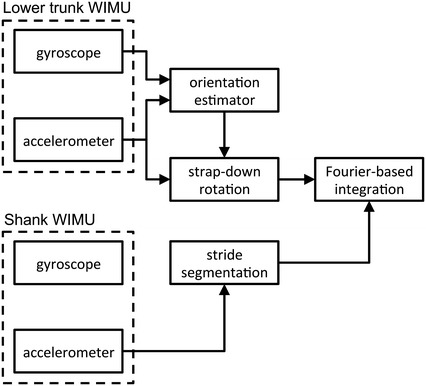
Block diagram of the proposed method for drift-free Fourier-based integration of gait acceleration data (Method A, see text)

Figure 4 shows the block diagram of the proposed method. In order to evaluate important factors that may vary in different approaches to 3D displacement estimation, a set of variants to the proposed algorithm were also tested. They were based on different implementations of strap-down rotation, stride segmentation (when needed) and integration, Table 1.

**Table 1:** Tested methods for 3D displacement estimation

Method	Strap-down quaternion	Sensor for stride segmentation	Integration method
Baseline method	OMCS-based	Shank gyroscope	Fourier-based
Method A	EKF-based	Shank gyroscope	Fourier-based
Method B	Initial value	Shank gyroscope	Fourier-based
Method C	EKF-based	Lower trunk accelerometer	Fourier-based
Method D	EKF-based	Shank gyroscope	Trapezoidal rule
Method E	EKF-based	None (high-pass filtering)	Trapezoidal rule

We tested the condition when the strap-down rotation was performed using the estimated quaternion (estimated rotation); the strap-down rotation was also performed using either the reference quaternion from the OMCS data (reference rotation) or the quaternion estimated during the rest period at the beginning of each walking trial (we called this option initial rotation). For each tested method of strap-down rotation, gait segmentation was based on the shank gyroscope and the integration was performed analytically to obtain 3D displacement estimation, leading to the baseline method (reference rotation), Method A (estimated rotation) and Method B (initial rotation). Method A (our proposed method) was implemented in two additional variants: lower trunk acceleration was used for gait segmentation, in place of the shank angular velocity, before carrying the Fourier-based integration (Method C); moreover, numerical integration was used, in place of the Fourier-based integration, in a situation when the shank angular velocity was used for gait segmentation (Method D). The numerical integration method was based on choosing the integration interval and the rule of integration as proposed in [9]. Stride time and stride time variability were computed for Method A and Method C. Finally, the displacements were computed following the standard approach of forward–backward filtering the acceleration signals via a second-order high-pass Butterworth filter with adapted cut-off frequency values, in preparation for their numerical integration (Method E). In accordance with the guidelines discussed in [11], we used a cut-off of 0.5 Hz (ML component) and 1 Hz (VT and AP components).Numerical integration was implemented as proposed in [9].

### Performance assessment and statistical tests

Steady-state locomotion strides were considered for performing the statistical analysis; hence, the walking data from the first and last 30-s periods of recording were discarded. For each walking trial, we computed the RMSD between the reference and the estimated Euler angles (angle RMSD), which were derived from the corresponding quaternion using standard conversion formulae. For each condition of walking speed, mean difference and upper and lower limit of agreement (mean difference ± 1.96 SD of differences) were computed for each component of displacement obtained from OMCS and IMU data. Methods A through to E, including the baseline method, were tested using the mean difference (MD) and the width of the limits of agreement (LA), namely the difference between the upper and lower limit. Scatter plots were also produced to visualize differences between data obtained using Method A and OMCS data against their mean [24].

## Results

The accuracy of the EKF in determining the orientation of the ULF relative to the GGF was determined by analyzing the angle RMSD, whose statistics, in terms of mean value ± standard deviation (SD)are reported in Table 2.

**Table 2:** Results of RMSD analysis (orientation), mean ± standard deviation (SD)

Speed, km/h	Roll, °	Pitch, °	Yaw, °
3	0.6 ± 0.2	0.8 ± 0.5	3.2 ± 1.5
4	0.5 ± 0.1	0.5 ± 0.2	2.8 ± 1.6
5	0.6 ± 0.2	0.9 ± 0.3	2.6 ± 1.9
6	0.8 ± 0.2	1.1 ± 0.5	2.7 ± 1.9
7	1.0 ± 0.4	1.2 ± 0.7	3.1 ± 2.3

The angular range of motion and the peak-to-trough values of the displacement in each direction, computed from the OMCS data and averaged across subjects, are reported in Tables 3, 4, for each condition of walking speed (mean value ± SD).

**Table 3:** Angular range of motion, mean ± SD

Speed, km/h	Roll, °	Pitch, °	Yaw, °
3	09.3 ± 2.1	7.3 ± 1.5	16.0 ± 3.4
4	09.4 ± 2.9	7.6 ± 3.3	12.9 ± 1.7
5	10.4 ± 3.5	6.9 ± 1.6	13.9 ± 4.0
6	11.6 ± 4.0	7.6 ± 1.4	16.1 ± 6.2
7	13.3 ± 4.0	8.7 ± 1.3	19.5 ± 3.8

**Table 4:** Peak-to-trough values, mean ± SD

Speed, km/h	ML direction, mm	VT direction, mm	AP direction, mm
3	60 ± 13	16 ± 6	28 ± 8
4	47 ± 8	26 ± 7	25 ± 6
5	39 ± 9	35 ± 7	24 ± 5
6	36 ± 8	44 ± 10	21 ± 5
7	34 ± 8	52 ± 15	20 ± 4

The stride time and the stride time variability for either Method A or Method C, averaged across subjects, are reported in Table 5, for each condition of walking speed (mean value ± SD).

**Table 5:** Stride time and stride time variability for Method A and Method C, mean ± SD

Speed, km/h	Shank gyroscope (Method A)		Lower trunk accelerometer (Method C)	
	Stride time, s	Stride time variability, %	Stride time, s	Stride time variability, %
3	1.32 ± 0.06	2.26 ± 0.63	1.32 ± 0.06	2.97 ± 1.02
4	1.13 ± 0.04	1.68 ± 0.74	1.13 ± 0.04	1.77 ± 0.66
5	1.02 ± 0.04	1.36 ± 0.43	1.02 ± 0.04	1.39 ± 0.38
6	0.94 ± 0.04	1.26 ± 0.30	0.94 ± 0.04	1.30 ± 0.30
7	0.87 ± 0.05	1.47 ± 0.45	0.87 ± 0.05	1.70 ± 0.68

**Fig. 5:**
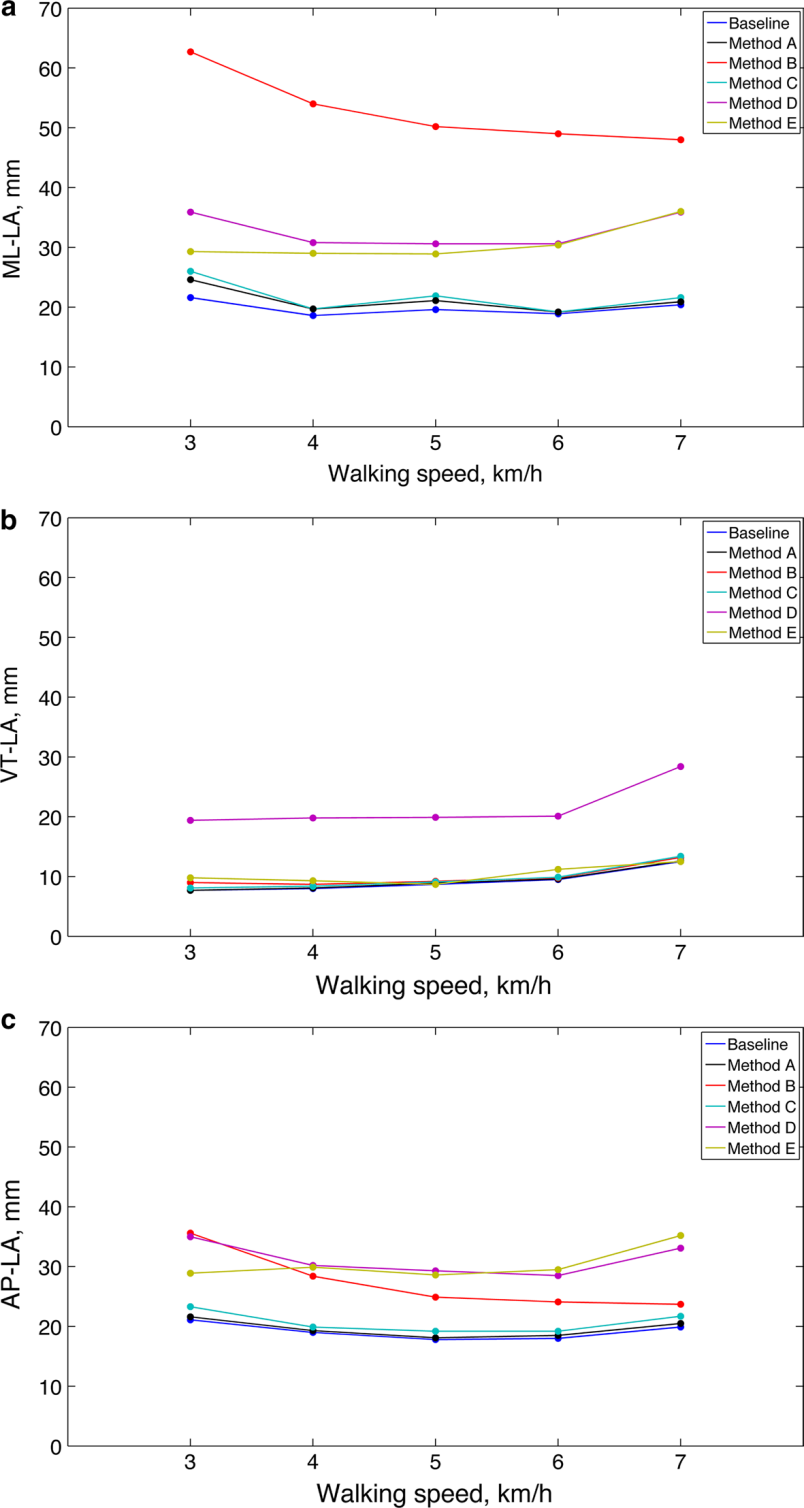
Width of limits of agreement for each component of displacement and tested method, as a function of walking speed: **a** ML component; **b** VT component; **c** AP component. For each walking speed, data from all subjects were considered in computing the desired statistics

MD and LA values were computed for each direction of displacement and tested method as a function of walking speed; since the values of MD were always less than ±0.1 mm (baseline, Method A, B and C) and ±0.6 mm (Method D and E), results are reported in Fig. 5 only for the values of LA.

**Fig. 6:**
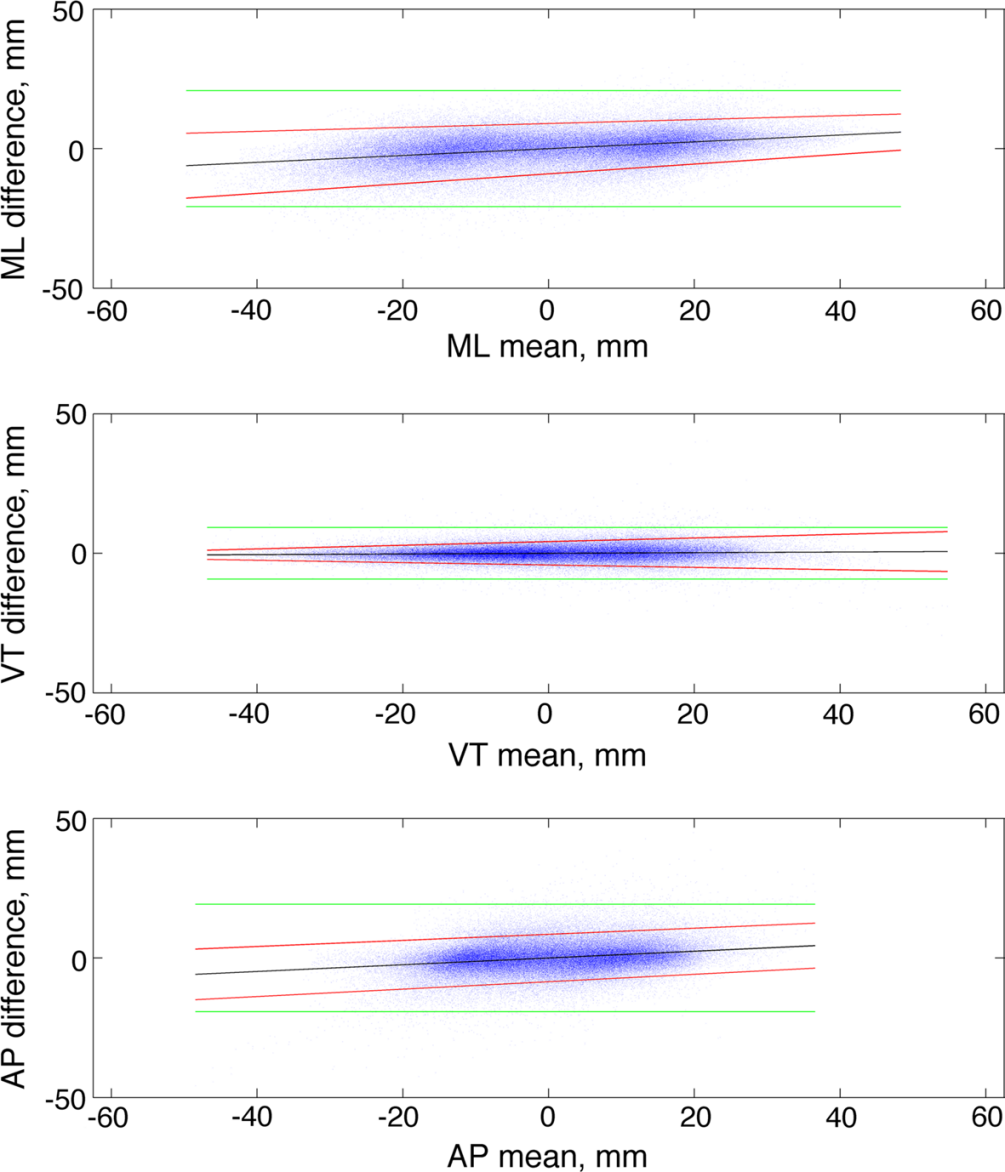
Bland Altman style plots showing the difference between OMCS and IMU sensors as a function of the mean of the two methods, for each component of displacement. Each plot is based on the samples collected for all subjects and conditions of walking speed. The *line* fitted to the plotted data is reported in *blue*. The upper and lower limits of agreement based on the regression approach described in [24] are reported in *red*; the *green lines* represent the constant limits of agreement computed without the regression-based correction

Finally, scatter plots of the difference between Method A and OMCS over their mean value are shown in Fig. 6. A slight tendency is observed for differences being increasingly positive with increasing mean value of the displacement; hence, compared to OMCS data, the proposed method slightly overestimated and underestimated for respectively negative and positive values of the displacement assessed in the global frame; the bias, computed by the mean difference, was less than 0.02 mm for all displacement components. Moreover, a slight tendency is observed for the spread of the differences to vary over the measurement range, which may produce non-constant LAs. In the attempt to refine the LA computation for the data in Fig. 6, the regression approach for non-uniform differences proposed in [24] was applied; the correction equations needed to compute the LA widths are reported in Table 6.

**Table 6:** Equations for computing LA widths for each displacement direction

	Upper and lower limits of agreement
ML direction	*D* = 0.1227 *A* − 0.0104 ± 2.46 (−0.0214 *A* + 3.6706)
VT direction	*D* = 0.0219 *A* − 0.0018 ± 2.46 (−0.0219 *A* + 1.7067)
AP direction	*D* = 0.1209 *A* − 0.0112 ± 2.46 (−0.0049 *A* + 3.4541)

*D*, difference between Method A and OMCS, mm; *A*, average value of Method A and OMCS, mm

For each displacement component, a representative value of the LA width was finally obtained by taking the average of the widths that were computed over a range of measured displacements covering 95 % of the values measured in our dataset. The following values were obtained: 18.1 mm (ML component), 8.3 mm (VT component) and 17.0 mm (AP component). In the absence of the regression-based correction, the LA widths were: 20.8 mm (ML component), 9.3 mm (VT component) and 19.2 mm (AP component).

## Discussion

The accuracy of orientation determination using the lower trunk IMU was especially good for roll and pitch angles at every walking speed. Yaw estimates were characterized by a higher uncertainty, which was reflected in higher and more dispersed angle RMSD values, Table 2. In our implementation, we did not use any sensing modality that could help stabilize the yaw estimate, such as the WIMU magnetic sensor, so as to comply with the experimental setups used in recent studies concerning the kinematics of pelvic motion [33, 34]. Moreover, each walking trial lasted 2 min, a time interval where gyro bias is shown to affect slightly the accuracy of orientation estimation [26]. The angular range of motion was assessed using reference OMCS data (Table 3) and was found to be consistent with findings from the literature, e.g., [34].

It is noted that an accelerometer measures specific force, namely the composition of two components, gravity acceleration and linear acceleration, which is caused by changes in velocity of the body part during motion. Although this work did not include anatomical calibration of the WIMU positioning, care was taken to align the WIMU axes to the anatomical axes. The lower trunk WIMU, in particular, might be tilted in several ways due to lumbar lordosis, postural alignment, or inaccuracy in mounting the device. Therefore, it is important to mathematically correct the data in order to assess linear acceleration in a horizontal-vertical coordinate system [35]. This tilt correction was always carried out during the rest period at the beginning of each walking trial. However, it is important to make corrections continuously during walking, so as to compensate, e.g., the gross postural changes of a subject. In dynamic conditions, the tilt cannot be corrected without a gyroscope tracking the changes of orientation, which was the operating mode of all methods apart from Method B.

The performance of the tested methods were analyzed in terms of the LA values, since the MD values were always small and negligible for all practical purposes, Fig. 5. For estimating the horizontal components of displacement, Method B was found to agree with the OMCS reference to a much lesser extent than all other methods, especially along the ML direction; on the contrary, the LA values were comparable along the vertical direction and were even better than Method D. Since the reference quaternion was based on the OMCS data, the baseline method can be considered the *best* approach for performing tilt correction in dynamic conditions. In general, the EKF performed very well when estimating the orientation; however, since the angular range of motion was not too large (Table 3), the performances achieved with and without tilt correction were not significantly different.

Different methods for detecting the beginning of each stride were tested. The shank is considered one of the best locations for a gyroscope to perform gait segmentation [28, 36], even in pathologic conditions, where strides can be identified with high robustness and good accuracy [37]. On the other hand, trunk accelerometry has been widely investigated for gait segmentation purposes [38]. Despite its widespread use in studies of human walking in pathologic conditions [17, 39], some doubt is cast upon its effectiveness [40]. Since this study focused on treadmill walking of healthy subjects, both methods were appropriate. From inspection of the results reported in Table 5, using the lower trunk accelerometer in place of the shank gyroscope led to slightly higher values of stride time variability.

It should be noted that the small variation of the gait period could be an argument to restrain the use of Fourier harmonic analysis [13]. In the present case, Fourier interpolation is applied to accurately model gait signals (i.e., the linear acceleration) over each detected stride-cycle, and the identified trigonometric polynomials are then used to approximate the integral of the gait signals over this same stride-cycle. The stride-by-stride analytical integration of strap-down rotated accelerations allowed highly accurate displacement estimation. A closer agreement with OMCS data was found for baseline, Method A and Method C, which are all based on analytical integration, as compared with methods based on numerical integration (Method D and Method E). In short, analytical integration performed better than numerical integration: the LA values increased by about 70 % when the integration was performed numerically than when it was analytical (horizontal components of displacement).The only exception was Method E (vertical component of displacement), whose LA values were similar to those achieved by methods based on analytical integration. The integration was carried over time intervals of limited duration (on average, the stride times were 1.32 s at the lowest walking speed and 0.87 s at the highest walking speed, Table 4); however, the integration drift is known to affect computation accuracy even for short integration intervals [41]. High-pass filtering with adapted cut-offs was shown to be very effective against low-frequency drifts in experiments on horses trotting overground [11, 42]. However, during treadmill walking, walkers are subject to a horizontal swaying that usually occurs over time scales of several strides and is not periodic. It is likely that the piecewise-constant trend removal scheme used in the proposed method is more effective against slow non-periodic components of motion, as compared with methods having lower temporal resolution, e.g., high-pass filtering.

Within the limits of the present investigation, the LA values achieved using Method A (the proposed method) do not seem to change significantly with the walking speed. The Bland–Altman scatter plots in Fig. 6 were computed by taking all samples of OMCS and inertial sensors collected for each subject and condition of walking speed. The regression approach for non-uniform differences proposed in [24] allowed accounting for the slight systematic differences between Method A and OMCS, and to establish average LA widths across the measurement range of pelvic displacements in this study. The regression-based correction helped avoid overestimation of the lower and upper limits of agreement, otherwise occurring in the case they were assumed constant over the measurement range.

In conclusion, the proposed method achieved, approximately, LAs of 8 mm (SD = 2 mm) for the VT component and of 18 mm (SD = 4.5 mm) for the ML and AP components, in the range of walking speeds from 3 to 7 km/h. LAs of similar magnitude were reported for horses trotting overground in [11]; moreover, the SD values were close to the RMS error reported for OMCS-based marker localization [43]. Agreement as a percentage of the range of motion (see Table 5) was found, approximately, in the range 25–50 % (VT component), 40–60 % (ML component) and 75–100 % (AP component). For the two components with higher range of motion, it is noted that the agreement of the proposed method with OMCS was worse in the ML direction than in the VT direction. An explanation of this fact is that ML acceleration was smaller compared with VT acceleration, and so more vulnerable to noise and signal artifacts.

## Conclusions

A method was developed in this paper to estimate the 3D displacement of an IMU positioned on the human body and subject to conditions of cyclical motion. The method is based on the analytical integration of stride-by-stride linear acceleration estimates using the Fourier coefficients that are obtained when the stride data are submitted to Fourier harmonic analysis. The validation study was carried out with the IMU attached to the level of the fifth lumbar vertebra when healthy subjects were walking on a treadmill at different speeds from 3 km/h (slow gait) to 7 km/h (fast gait).

We showed that, in order to exploit the best agreement with OMCS data, the following conditions are to be met: (a) the measured specific force is strap-down rotated using an estimate of the orientation from the body frame to the absolute reference frame and compensated for gravity; (b) the beginning and end of each stride are detected by using either the IMU sensors or any other available source, e.g., a shank gyroscope; (c) the linear acceleration stride data are submitted to analytical integration instead of numerical integration.

We are confident that the proposed method can be successfully applied in a wider context than outlined in this paper for the following reasons: (a) Fourier harmonic analysis is routinely adopted for HR-based gait assessment [15, 16, 19]; (b) IMU-based orientation determination is fairly accurate for different IMU placement sites and highly dynamic motor tasks [26]; (c) stride segmentation using inertial sensors is robust even for pathologic or geriatric gait [33, 34, 37]. Our current research agenda concerns the application of the proposed method for tracking the lower trunk and other anatomical points, even in conditions of pathologic and geriatric gait.
